# Stem Cell Niches for Skin Regeneration

**DOI:** 10.1155/2012/926059

**Published:** 2012-06-03

**Authors:** Victor W. Wong, Benjamin Levi, Jayakumar Rajadas, Michael T. Longaker, Geoffrey C. Gurtner

**Affiliations:** ^1^Department of Surgery, Oregon Health & Science University, 3181 Southwest Sam Jackson Park Road, Portland, OR 97239, USA; ^2^Hagey Laboratory for Pediatric Regenerative Medicine, Department of Surgery, Stanford University, 257 Campus Drive, Stanford, CA 94305, USA; ^3^Department of Surgery, Plastic and Reconstructive Surgery Division, Division of Burn Surgery, University of Michigan Health Systems, 1500 East Medical Center Drive, Ann Arbor, MI 48104, USA; ^4^The Biomaterials and Advanced Drug Delivery (BioADD) Laboratory, Stanford University, 300 Pasteur Drive, Grant Building, Room S380, Stanford, CA 94305, USA

## Abstract

Stem cell-based therapies offer tremendous potential for skin regeneration following injury and disease. Functional stem cell units have been described throughout all layers of human skin and the collective physical and chemical microenvironmental cues that enable this regenerative potential are known as the stem cell niche. Stem cells in the hair follicle bulge, interfollicular epidermis, dermal papillae, and perivascular space have been closely investigated as model systems for niche-driven regeneration. These studies suggest that stem cell strategies for skin engineering must consider the intricate molecular and biologic features of these niches. Innovative biomaterial systems that successfully recapitulate these microenvironments will facilitate progenitor cell-mediated skin repair and regeneration.

## 1. Introduction

Skin serves as the interface with the external world and maintains key homeostatic functions throughout life. This regenerative process is often overlooked until a significant exogenous and/or physiologic insult disrupts our ability to maintain skin homeostasis [1]. Complications of normal repair often result in chronic wounds, excessive scarring, or even malignant transformation, cutaneous diseases that contribute substantially to the global health burden [2, 3]. As human populations prone to inadequate healing (such as the aged, obese, and diabetics) continue to expand, novel therapies to treat dysfunctional skin repair and regeneration will become more critical.

Tissue regeneration has been demonstrated in multiple invertebrate and vertebrate species [4]. In humans, even complex tissues can regenerate without any permanent sequelae, such as liver, nerves, and skin. Although the typical result after significant organ injury is the formation of scar, regeneration after extensive skin and soft tissue trauma has been reported, most notably after digit tip amputation [5]. It is well accepted that human skin maintains the ability to regenerate; the question for researchers and clinicians is how to harness this potential to treat cutaneous injury and disease.

The integumentary system is a highly complex and dynamic system composed of myriad cell types and matrix components. Numerous stem cell populations have been identified in skin and current research indicates that these cells play a vital role in skin development, repair, and homeostasis [1, 6, 7]. In general, stem cells are defined by their ability to self-renew and their capacity to differentiate into function-specific daughter cells. These progenitor cells have been isolated from all skin layers (epidermis, dermis, hypodermis) and have unique yet complimentary roles in maintaining skin integrity. The promise of regenerative medicine lies in the ability to understand and regulate these stem cell populations to promote skin regeneration [4].

## 2. Stem Cells in Wound Healing

Wound healing is a highly regulated process that is thought to be mediated in part by stem cells [8, 9]. This has prompted researchers to examine the use of stem cells to augment skin repair following injury. Preclinical studies have suggested that the secretion of paracrine factors is the major mechanism by which stem cells enhance repair [10, 11]. Consistent with this hypothesis, conditioned media from mesenchymal stem cells (MSCs) have been shown to promote wound healing via activation of host cells [11, 12]. Clinical studies have suggested that topical delivery of MSCs may improve chronic wound healing [13–15] and multiple groups have demonstrated the benefit of using recombinant cytokines (many of which are known to be secreted by stem cells) in patients with recalcitrant wounds [16]. However, more research is needed to determine the mechanisms by which stem cell therapies might improve wound healing in humans.

For example, the extent of stem cell engraftment and differentiation following topical delivery remains unclear. In one study, bone-marrow-derived allogeneic MSCs injected into cutaneous wounds in mice were shown to express keratinocyte-specific proteins and contributed to the formation of glandular structures after injury [17]. Although long-term engraftment was poor (only 2.5% of MSCs remained engrafted after four weeks), levels of secreted proangiogenic factors were greater in MSC-treated wounds. Our laboratory has demonstrated that local injection of allogeneic MSCs improved early wound closure in mice but that injected MSCs contributed to less than 1% of total wound cells after four weeks [18]. Taken together, these studies suggest that the benefits observed with stem cell injections are the result of early cytokine release rather than long-term engraftment and differentiation.

**Figure 1:**
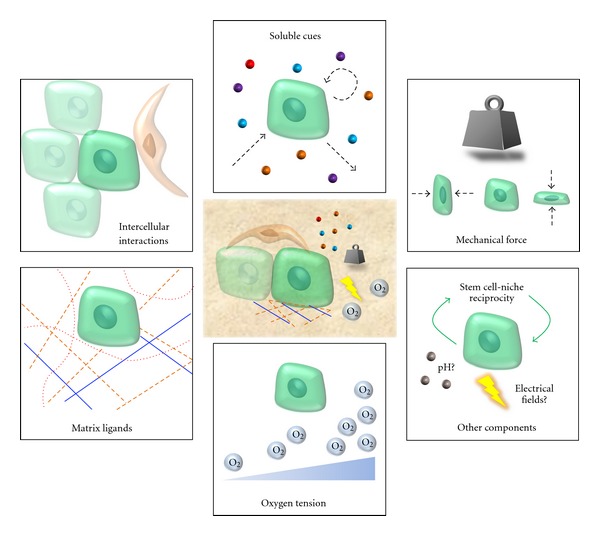
Potential components of the skin stem cell niche. Features common to skin stem cell niches include dynamic regulation of matrix ligands, intercellular interactions, and biochemical gradients in the appropriate three-dimensional contexts. Engineered biomaterials offer the potential to effectively pattern and regulate these biologic cues on increasingly precise time and space scales.

One potential reason for the transient presence of exogenous stem cells is the absence of proper contextual cues after cells are delivered into the wound. The dynamic microenvironment, or niche, of stem cells is responsible for regulating their “stem-like” behavior throughout life [19, 20]. This niche is comprised of adjacent cells (stem and nonstem cells), signaling molecules, matrix architecture, physical forces, oxygen tension, and other environmental factors (Figure 1). A useful analogy is the “seed versus soil” paradigm in which seeds (stem cells) will only thrive in the proper chemical and physical soil environment (wound bed) [4]. Clearly, we need to better define what these niches are and how they dictate cell behavior to fully realize the potential of progenitor cell therapies.

## 3. The Epidermal Stem Cell Niche

The epidermis is comprised of at least three major stem cell populations: the hair follicle bulge, the sebaceous gland, and the basal layer of interfollicular epithelium [21]. Because these subpopulations are responsible for regulating epithelial stratification, hair folliculogenesis, and wound repair throughout life [22], the epidermis has become a model system to study regeneration. Elegant lineage tracing and gene mapping experiments have elucidated key programs in epidermal homeostasis. Specifically, components of the wingless-type (Wnt)/*β*-catenin, sonic hedgehog (Shh), and transforming growth factor (TGF)-*β*/bone morphogenetic protein (BMP) pathways appear to be particularly relevant to epidermal stem cell function [1, 22, 23]. Microarray analyses have even indicated that hair follicle stem cells share some of the same transcriptomes as other tissue-specific stem cells [24], suggesting that conserved molecular machinery may control how environmental stimuli regulate the stem cell niche [25].

 Epithelial stem cells from the bulge, sebaceous gland, and basal epithelium have common features, including expression of K5, K14, and p63, and their intimate association with an underlying basement membrane (BM) [26]. These cells reside in the basal layer of stratified epithelium and exit their niche during differentiation [26]. This process is mediated in part by BM components such as laminin and cell surface transmembrane integrins that control cell polarity, anchorage, proliferation, survival, and motility [27, 28]. Epithelial progenitor cells are also characterized by elevated expression of E-cadherin in adherens junctions and reduced levels of desmosomes [29], underscoring the importance of both extracellular and intercellular cues in stem cell biology.

In addition to complex intraepithelial networks, signals from the dermis (e.g., periodic expression of BMP2 and BMP4) are thought to regulate epithelial processes [30]. Dermal-derived stem cells may even differentiate into functional epidermal melanocytes [31], suggesting that mesenchymal-epithelial transitions may underlie skin homeostasis, as has been shown in hepatic stem cells [32]. Recently, it has been demonstrated that irreversibly committed progeny from an epithelial stem cell lineage may be “recycled” and contribute back to the regenerative niche [33], further highlighting the complexity of the epidermal regeneration.

## 4. The Dermal Niche

In contrast to the highly cellular nature of the epidermis, the dermis is composed of a heterogeneous matrix of collagens, elastins, and glycosaminoglycans interspersed with cells of various embryonic origin. Recent studies suggest that a cell population within the dermal papilla of hair follicles may function as adult dermal stem cells. This dermal unit contains at least three unique populations of progenitor cells differentiated by the type of hair follicle produced and the expression of the transcription factor Sox2 [34]. Sox2-expressing cells are associated with Wnt, BMP, and fibroblast growth factor (FGF) signaling whereas Sox2-negative cells utilize Shh, insulin growth factor (IGF), Notch, and integrin pathways [35, 36]. Skin-derived precursor (SKP) cells have also been isolated from dermal papillae and can be differentiated into adipocytes, smooth myocytes, and neurons in vitro [37, 38]. These cells are thought to originate in part from the neural crest and have been shown to exit the dermal papilla niche and contribute to cutaneous repair [39].

 Researchers have also demonstrated that perivascular sites in the dermis may act as an MSC-like niche in human scalp skin [40]. These perivascular cells express both NG2 (a pericyte marker) and CD34 (an MSC and hematopoietic stem cell marker) and are predominantly located around hair follicles. Perivascular MSC-like cells have been shown to protect their local matrix microenvironment via tissue-inhibitor-of-metalloproteinase (TIMP-) mediated inhibition of matrix metalloproteinase (MMP) pathways, suggesting the importance of the extracellular matrix (ECM) niche in stem cell function [41]. Interestingly, even fibroblasts have been shown to maintain multilineage potential in vitro and may play important roles in skin regeneration that have yet to be discovered [42, 43].

## 5. The Adipose Niche

The ability to harvest progenitor cells from adipose tissues is highly appealing due to its relative availability (obesity epidemic in the developed world) and ease of harvest (lipoaspiration). Secreted cytokines from adipose-derived stem cells (ASCs) have been shown to promote fibroblast migration during wound healing and to upregulate VEGF-related neovascularization in animal models [44]. ASCs have even been harvested from human burn wounds and shown to engraft into cutaneous wounds in a rat model [45]. Although these multipotent cells have only been relatively recently identified, they exhibit significant potential for numerous applications in skin repair [46].

ASCs are often isolated from the stromal vascular fraction (SVF) of homogenized fat tissue. These multipotent cells are closely associated with perivascular cells and maintain the potential to differentiate into smooth muscle, endothelium, adipose tissue, cartilage, and bone [47, 48]. Researchers have attempted to recreate the ASC niche using fibrin matrix organ culture systems to sustain adipose tissue [49]. Using this in vitro system, multipotent stem cells were isolated from the interstitium between adipocytes and endothelium, consistent with the current hypothesis that ASCs derive from a perivascular niche.

Detailed immunohistological studies have demonstrated that stem cell markers (e.g., STRO-1, Wnt5a, SSEA1) are differentially expressed in capillaries, arterioles, and arteries within adipose tissue, suggesting that ASCs may actually be vascular stem cells at diverse stages of differentiation [50]. Adipogenic and angiogenic pathways appear to be concomitantly regulated and adipocytes secrete multiple cytokines that induce blood vessel formation including vascular endothelial-derived growth factor (VEGF), FGF2, BMP2, and MMPs [51, 52]. Additionally, cell surface expression of platelet-derived growth factor receptor *β* (PDGFR*β*) has been linked to these putative mural stem cells [53]. Reciprocal crosstalk between endothelial cells and ASCs may regulate blood vessel formation [54] and immature adipocytes have been shown to control hair follicle stem cell activity through PDGF signaling [55]. Taken together, these studies indicate that the ASC niche is intimately associated with follicular and vascular homeostasis but further studies are needed to precisely define its role in skin homeostasis [48].

## 6. Engineering Niches through Biomaterials

Strategies to recapitulate the complex microenvironments of stem cells are essential to maximize their therapeutic potential. Biomaterial-based approaches can precisely regulate the spatial and temporal cues that define a functional niche [56]. Sophisticated fabrication and bioengineering techniques have allowed researchers to generate complex three-dimensional environments to regulate stem cell fate. As the physicochemical gradients, matrix components, and surrounding cells constituting stem cell niches in skin are further elucidated (Table 1), tissue engineered systems will need to be increasingly scalable, tunable, and modifiable to mimic these dynamic microenvironments [57–61]. A detailed discussion of different biomaterial techniques for tissue engineering is beyond the scope of this paper, but we refer to reader to several excellent papers on the topic [62–70].

 One matrix component thought to regulate interactions between hair follicle stem cells and melanocyte stem cells is the hemidesmosomal collagen XVII [71]. Collagen XVII controls their physical interactions and maintains the self-renewal capacity of hair follicles via TGF-*β*, indicating that biomaterial scaffolds containing collagen XVII may be necessary for stem cell-mediated hair follicle therapies. Another matrix component implicated in the hair follicle niche is nephronectin, a protein deposited into the underlying basement membrane by bulge stem cells to regulate cell adhesion via *α*8*β*1 integrins [72]. Hyaluronic acid fibers have been incorporated into collagen hydrogels to promote epidermal organization following keratinocyte seeding [73], and in vitro studies have demonstrated the critical role of collagen IV in promoting normal epithelial architecture when keratinocytes are grown on fibroblast-populated dermal matrices [74]. These studies collectively suggest that tissue engineered matrices for skin regeneration will need to recapitulate the complex BM-ECM interactions that define niche biology [75].

The role of MSCs in engineering skin equivalents has been studied using either cell-based or collagen-based dermal equivalents as the scaffolding environment [76]. When these constructs were grown with keratinocytes in vitro, only the collagen-based MSCs promoted normal epidermal and dermal structure, leading the authors to emphasize the “necessity of an instructive biomaterial-based scaffold to direct stem cell differentiation, proliferation, paracrine activity [and] ECM deposition [76].” Our laboratory has reported that MSCs seeded into dermal-patterned hydrogels maintain greater expression of the stem cell transcription factors Oct4, Sox2, and Klf4 as compared to those grown on two-dimensional surfaces [18]. MSCs seeded into these niche-like scaffolds also exhibited superior angiogenic properties compared to injected cells [18], indicating that stem cell efficacy may be enhanced with biomaterial strategies to recapitulate the niche. Another study demonstrated that ASC delivery in natural-based scaffolds (dermis or small intestine submucosa) resulted in improved wound healing compared to gelatin-based scaffolds, suggesting the importance of “biologically accurate” architecture for stem cell delivery [77].

Researchers have developed novel three-dimensional microfluidic devices to study perivascular stem cell niches in vitro [78]. For example, MSCs seeded with endothelial cells in fibrin gels were able to induce neovessel formation within microfluidic chambers through *α*6*β*1 integrin and laminin-based interactions. Fibrin-based gels have also been used to study ASC and endothelial cell interactions in organ culture [49] and to control ASC differentiation in the absence of exogenous growth factors, demonstrating the importance of the three-dimensional matrix environment in regulating the ASC niche [79]. These studies indicate that the therapeutic use of ASCs in skin repair will likely be enhanced with biomaterial systems that optimize these cell-cell and cell-matrix contacts.

Finally, it must be recognized that the wound environment is exceedingly harsh and often characterized by inflammation, high bacterial loads, disrupted matrix, and/or poor vascularity. In this context, it should not be surprising that injection of “naked” stem cells into this toxic environment does not produce durable therapeutic benefits. Our laboratory has shown that the high oxidative stress conditions of ischemic wounds can be attenuated with oxygen radical-quenching biomaterial scaffolds that also deliver stem cells [80]. Other researchers have shown that oxygen tension, pH levels, and even wound electric fields may influence stem cell biology, suggesting that the future development of novel sensor devices will allow even finer control of chemical microgradients within engineered niches [70, 81]. It is also important to acknowledge that current research on niche biology has been performed largely in culture systems or rodent models, findings that will need to be rigorously confirmed in human tissues before clinical use.

**Figure 2:**
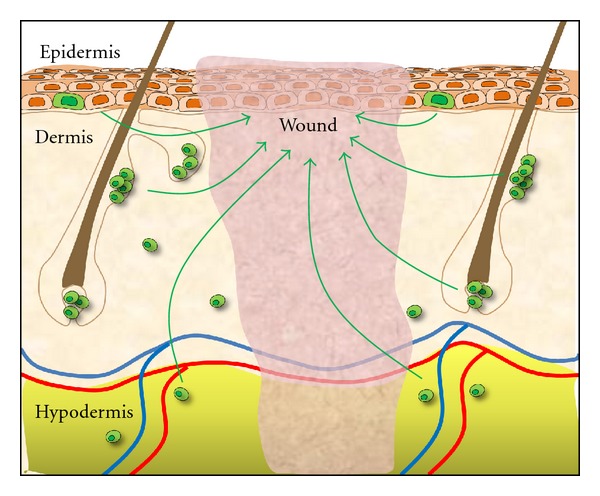
Locally derived skin stem cells may harbor the potential to regenerate skin. Stem cells populations have been identified in various niches throughout the skin, including the epidermal stem cell in the hair follicle bulge, sebaceous glands, and interfollicular epidermis. Dermal stem cells may exist in the dermal papilla or dermal extracellular matrix. Adipose-derived stem cells appear to be intimately associated with the perivascular space.

As interdisciplinary fields such as material science, computer modeling, molecular biology, chemical engineering, and nanotechnology coordinate their efforts, multifaceted biomaterials will undoubtedly be able to better replicate tissue-specific niche environments. Recent studies suggest that the cells necessary for skin regeneration are locally derived [5], indicating that adult resident cells alone may have the ability to recreate skin (Figure 2). Thus, the ability to engineer the proper environment for skin stem cells truly has the potential to enable regenerative outcomes. We believe that next-generation biomaterial scaffolds will not only passively deliver stem cells but also must actively modify the physicochemical milieu to create a “therapeutic” niche.

## 7. Conclusion

Current research indicates that skin regeneration is highly dependent upon interactions between resident progenitor cells and their niche. These microenvironmental cues dictate stem cell function in both health and disease states. Early progress has been made in elucidating skin compartment-specific niches but a detailed understanding of their molecular and structural biology remains incomplete. Biomaterials will continue to play a central role in regenerative medicine by providing the framework upon which to reconstruct functional niches. Future challenges include the characterization and recapitulation of these dynamic environments using engineered constructs to maximize the therapeutic potential of stem cells.

## Figures and Tables

**Table 1:** Skin-specific stem cells and putative features of their niche.

In situ location	Epidermal stem cell	Dermal stem cell	Adipose stem cell
	Hair follicle bulge	Dermal papilla	Perivascular/vascular
Signaling pathways	Wnt	Wnt	VEGF
	*β*-catenin	Timp	PPAR*γ*
	Shh	BMPs	FGF2
	TGF*β*	FGF	MMPs
	BMPs	Shh	PDGF
	p63	IGF	
		Notch	

Surface and structural proteins	K5	NG2	CD29
	K14	CD34	CD44
	K15	CD44	CD73
	E-cadherin	CD54	CD90
	LGR5	CD73	CD105
	LGR6	CD90	CD166
	CD29	CD105	PDGFR*β*
	CD34	CD133	Integrin *α*6*β*1
	CD49f	CD271	
	CD117		
	CD200		

Potential matrix components	Laminin		Fibrin
	Collagen IV		Collagen I
	Collagen XVII		
	Nephronectin		

Wnt: wingless-type; Shh: sonic hedgehog; BMP: bone morphogenetic protein; TGF*β*: transforming growth factor *β*, VEGF: vascular endothelial growth factor; FGF: fibroblast growth factor; IGF: insulin-like growth factor; PDGF: platelet-derived growth factor; PPAR*γ*: peroxisome proliferator-activated receptor *γ*; MMP: matrix metalloproteinase; Timp: tissue inhibitor of metalloproteinases.
